# Lipoprotein(a) is not related to markers of insulin resistance in pregnancy

**DOI:** 10.1186/1475-2840-12-138

**Published:** 2013-10-01

**Authors:** Jelena Todoric, Ammon Handisurya, Karoline Leitner, Juergen Harreiter, Gregor Hoermann, Alexandra Kautzky-Willer

**Affiliations:** 1 Laboratory of Gene Regulation and Signal Transduction, Departments of Pharmacology and Pathology, School of Medicine, University of California San Diego, 9500 Gilman Dr, La Jolla, CA 92093-0636, USA; 2 Department of Laboratory Medicine, Medical University of Vienna, Waehringer Guertel 18-20, 1090 Vienna, Austria; 3 Department of Internal Medicine III, Division of Nephrology and Dialysis, Medical University of Vienna, Waehringer Guertel 18-20, 1090 Vienna, Austria; 4 Department of Internal Medicine III, Division of Endocrinology and Metabolism, Medical University of Vienna, Waehringer Guertel 18-20, 1090 Vienna, Austria

**Keywords:** Lipoprotein(a), Pregnancy, Gestational diabetes mellitus, Insulin resistance

## Abstract

**Background:**

Dyslipidemia, a major risk factor for cardiovascular disease is a common finding in patients with type 2 diabetes and among women with gestational diabetes. Elevated levels of lipoprotein(a) [Lp(a)] are linked to increased risk of cardiovascular disease. However, its relationship with insulin resistance, type 2 diabetes and gestational diabetes is controversial and unproven. Here we aimed to clarify whether Lp(a) levels are associated with insulin sensitivity in pregnancy.

**Methods:**

Sixty-four women with gestational diabetes and 165 with normal glucose tolerance were enrolled in the study. Fasting Lp(a) serum levels were measured in all women at 24–28 weeks of gestation.

**Results:**

In pregnancy, there was no significant difference in serum Lp(a) concentrations between the two groups. Its level did not correlate with markers of insulin resistance (HOMA-IR), insulin sensitivity (HOMA-S%), pancreatic beta-cell function (HOMA-B%) and insulin sensitivity in dynamic conditions (OGIS). In addition, fasting glucose and insulin levels and those throughout an oral glucose tolerance test were independent of Lp(a) concentrations in our study group.

**Conclusions:**

Lp(a) levels in pregnant women do not differ with respect to the presence or absence of gestational diabetes. Although influenced by some components of the lipid profile, such as triglycerides and HDL-C, insulin resistance in pregnancy is not affected by Lp(a).

## Background

Lipoprotein(a) [Lp(a)] is a plasma lipoprotein that consists of an low-density lipoprotein cholesterol (LDL-C) particle and the glycoprotein apolipoprotein(a) covalently linked to apolipoprotein B100 of the LDL-C particle [1]. There are strong genetic and epidemiological data supporting a causal relationship between elevated Lp(a) concentrations and the development of atherosclerosis and cardiovascular disease (CVD) [2-4]. Type 2 diabetes (T2D) and CVD share common risk factors, including obesity, hyperinsulinemia and dyslipidemia [5]. Both diseases can also be prevented or treated more effectively if they are diagnosed early and there is substantial interest in identifying new risk factors involved in their development. Lipid abnormalities frequently observed in subjects with insulin resistance and T2D are hypertriglyceridemia, low high-density lipoprotein cholesterol (HDL-C), and an increased fraction of small, dense LDL-C particles [6]. However, previous reports regarding Lp(a) levels and T2D are conflicting [7]. Decreased Lp(a) levels in response to hyperinsulinemia [8,9] and a negative relationship between Lp(a) levels and the incidence of T2D have been reported previously [10,11]. In contrast, some studies have shown a strong positive association between T2D and elevated Lp(a) levels [12,13]. Similar Lp(a) concentrations in subjects with T2D and healthy controls have been reported in one study [14]. No studies so far have investigated the association between Lp(a) and insulin sensitivity parameters in gestational diabetes mellitus (GDM).

GDM, defined as glucose intolerance that begins or is first detected during pregnancy affects 7% of all pregnancies [15-17]. The majority of patients with GDM have beta- cell dysfunction associated with insulin resistance [18]. The atherogenic lipid profile is a common finding during the second half of pregnancy and the mechanisms underlying such alterations of lipid metabolism are not fully understood [19,20]. However, dyslipidemia during pregnancy may also be a pathological finding suggesting the development of metabolic syndrome. For example, elevated triglyceride (TG) levels have been reported in women with GDM [21] and were associated with diagnosis of GDM [22].

Thus, the goal of this study was to analyze the relationships between Lp(a) levels and parameters of glucose metabolism and insulin sensitivity in patients with GDM and healthy control women during pregnancy.

## Methods

### Study population

Women enrolled in this study were recruited from the outpatient clinic of the Division of Endocrinology and Metabolism at the Medical University of Vienna. Two hundred and twenty nine pregnant women of a similar age and body mass index (BMI) were recruited via universal screening for GDM that was performed with a 2 h, 75 g oral glucose tolerance test (OGTT) between the 24th and 28th weeks of gestation. Sixty- four women were diagnosed with GDM and 165 had a normal glucose tolerance (NGT). Most women were Caucasians (95,6%; 60 GDM and 159 NGT), whereas 1,7% were of African origin (1 GDM and 3 NGT) and 2,6% were Asian (3 GDM, 3 NGT). None of these women had a history of GDM or any history of impaired glucose tolerance. Fasting Lp(a) concentrations were measured in all women. All participants provided written informed consent, the study was conducted in accordance with the Declaration of Helsinki (2008) and approved by the ethics committee of the Medical University of Vienna. GDM was diagnosed following the recommendations of the American Diabetes Association [23].

### 75 g OGTT

A 75-g OGTT was performed after a 12 h overnight fast. Plasma glucose and insulin concentrations were measured before and 30, 60, 90, and 120 min after the ingestion of the glucose solution. Venous blood sera were obtained by centrifugation and analyzed immediately or frozen at −80°C until further analysis.

### Assays

Lp(a) was quantified using the turbidimetric LPALX assay (Roche) on a cobas c 701 analyzer (Roche) according to the recommendation of the manufacturer. The assay was independent of apolipoprotein(a) isoform size. Hormones, metabolic and kidney function parameters were measured via routine tests in a certified laboratory at the Department of Laboratory Medicine of the Medical University of Vienna.

### Data analysis

The trapezoidal rule was used to determine the area under the concentration curve of glucose, insulin and C-peptide (AUCg, AUCi and AUCcp, respectively) during the OGTT. The homeostasis model assessment (HOMA) was used to evaluate insulin resistance (HOMA-IR), insulin sensitivity indexes (HOMA-S%) and steady state beta-cell function (HOMA-B%). The HOMA Calculator software v2.2.2 (http://www.dtu.ox.ac.uk/index.php?maindoc=/homa/index.php) was utilized for these calculations [24]. During OGTT, dynamic insulin sensitivity was assessed from the oral glucose insulin sensitivity index (OGIS) [25].

### Statistical analyses

Data are presented as mean ± SE. Distributions of continuous variables were tested for normality using the Shapiro-Francia test, and, if appropriate, the natural log transformations of skewed variables were applied in subsequent analyses. Relationships between continuous variables were evaluated using Spearman rank correlation coefficients. Multiple linear regression models were constructed to examine factors that were associated with Lp(a) concentration. Two-way ANOVA was performed to evaluate differences between the groups. Statistical analyses were performed using SPSS software (SPSS 17.0, Chicago, IL). A *P* value of <0.05 was considered statistically significant.

## Results

**Figure 1:**
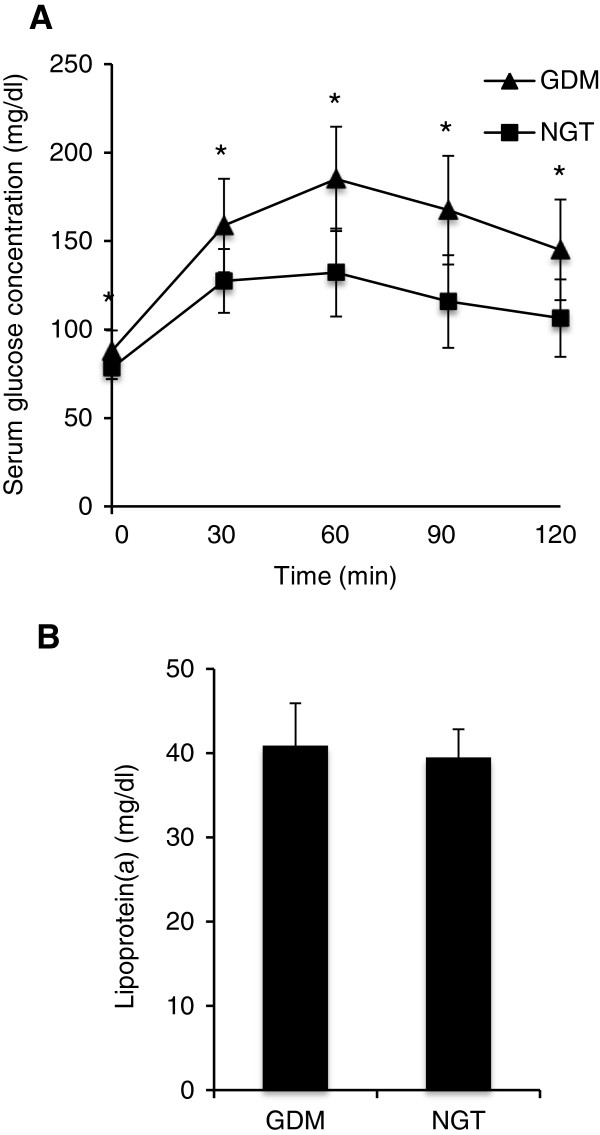
**Serum Lp(a) levels in GDM patients and healthy pregnant women. (A)** Serum glucose concentrations during OGTT. **(B)** Fasting Lp(a) serum levels in GDM and NGT women. Data are expressed as mean ± S.E. *, *P* < 0.001.

Characteristics of the study population are described in Table 1. As shown, patients with GDM had significantly higher fasting glucose, insulin, C-peptide, A1C, TG and HOMA-IR whereas HOMA-S%, the HDL-C and LDL-C levels were lower than in the control group (GDM vs NGT; P < 0.001, P = 0.008, P < 0.001, P < 0.001, P = 0.024, P = 0.002, P = 0.008, P = 0.005 and P = 0.017, respectively). HOMA-B% value was lower in the GDM women, but the difference to the control group just failed to reach statistical significance (P = 0.075). Moreover, GDM patients showed significantly higher levels of glucose at all time points during the OGTT (Figure 1A). AUCg, AUCi and AUCcp were significantly increased in women with GDM compared with healthy controls (GDM vs NGT; 18.6 ± 0.4 g/dl × 120 min vs 13.9 ± 0.2 g/dl × 120 min, P < 0.001; 11.6 ± 0.8 U/l × 120 min vs 8.2 ± 0.3 U/l × 120 min, P < 0.001 and 1177.5 ± 55.9 ng/ml × 120 min vs 940.2 ± 25.9 ng/ml × 120 min, P < 0.001, respectively). The OGIS showed lower values in patients with GDM compared with NGT women (470.4 ± 9.7 ml × min ^-1^ × m ^-2^ vs 525 ± 3.8 ml × min ^-1^ × m ^-2^, P < 0.001). The mean of age, BMI, systolic and diastolic blood pressure, C-reactive protein (CRP), total cholesterol, creatinine, estradiol and progesterone was similar for patients and controls.

**Table 1:** Baseline characteristics, metabolic and hormonal parameters in women with GDM and healthy controls

	**GDM**	**NGT**	***P***
	**n = 64**	**n = 165**	
Age, years	33.6 (0.7)	32.6 (0.4)	0.228
BMI, kg/m2	28.9 (0.7)	27.2 (0.4)	0.055
Parity	1.3 (0.2)	1.1 (0.1)	0.110
Systolic blood pressure (mmHg)	112.9 (1.2)	109.9 (0.9)	0.082
Diastolic blood pressure (mmHg)	70.4 (1.1)	69.7 (0.8)	0.664
Fasting glucose, mg/dl	87.4 (1.4)	78.4 (0.5)	**< 0.001**
Fasting insulin, mU/l	14.3 (0.9)	11.4 (0.5)	**0.008**
Fasting C-peptide, μg/l	2.5 (0.1)	1.6 (0.1)	**< 0.001**
A1C, %	5.2 (0.1)	4.8 (0.1)	**< 0.001**
HOMA-IR	1.8 (0.1)	1.4 (0.1)	**0.002**
HOMA-S%	72.4 (7.5)	92.1 (3.8)	**0.008**
HOMA-B%	151.2 (7.5)	166.7 (4.6)	0.075
Triglycerides, mg/dl	195.3 (8.3)	173.4 (6.5)	**0.024**
Total cholesterol, mg/dl	246.9 (5.7)	261.5 (3.7)	0.051
HDL cholesterol, mg/dl	68.9 (2.1)	75.7 (1.2)	**0.005**
LDL cholesterol, mg/dl	133.9 (5.3)	147.8 (2.8)	**0.017**
CRP, mg/dl	1.2 (0.3)	0.9 (0.6)	0.471
Creatinine, mg/dl	0.60 (0.01)	0.62 (0.01)	0.084
Estradiol, pg/ml	10113.2 (689.6)	11056.8 (447.8)	0.308
Progesterone, ng/ml	76.9 (6.9)	79.2 (3.5)	0.753

Variables are presented as mean ± S.E. The statistical significance of differences between the study groups was determined by ANOVA. The *P* values correspond to the differences between GDM and NGT women. Statistically significant *P* values are labeled with bold text.

**Figure 2:**
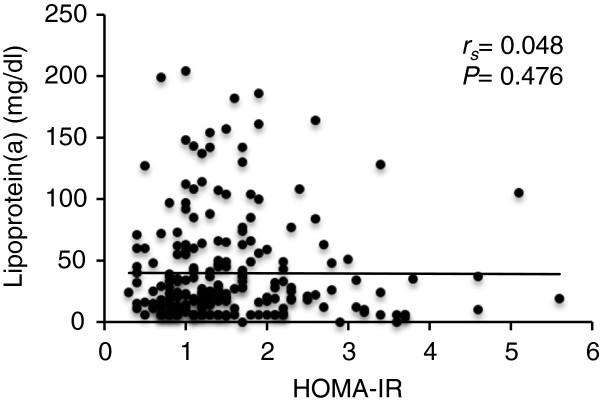
**Relationship between insulin resistance and Lp(a) in the whole study group.** Scatterplot represents the correlation between the Lp(a) and insulin resistance parameter HOMA-IR. *r*_*s*_, Spearman’s correlation coefficient.

The mean level of Lp(a) was 40,89 +/− 5,02 mg/dl and 39,47 +/− 3,37 mg/dl in patients with GDM and healthy women, respectively and there was no statistically significant difference in Lp(a) concentrations between the two groups (Figure 1B). Most of the variables traditionally linked to T2D including markers of insulin resistance, fasting insulin, HOMA-B%, HOMA-S% and HOMA-IR were not associated with the Lp(a) levels (Table 2, Figure 2). Serum Lp(a) levels showed a significant positive correlation with LDL-C and were negatively related to estradiol (Table 2; P = 0.022 and P = 0.019 respectively). After adjustment for age and BMI, both LDL-C and estradiol emerged as independent predictors of Lp(a) concentration in multiple linear regression analyses (t = 2.474, P = 0.014; t = −2.164, P = 0.032).

**Table 2:** Correlations of Lp(a) with metabolic and insulin sensitivity parameters in the whole study group

	***r***_***s***_	***P***
BMI	−0.075	0.291
Fasting glucose, mg/dl	−0.013	0.850
Fasting insulin, mU/l	0.038	0.570
Fasting C-peptide, μg/l	0.047	0.490
A1C, %	0.024	0.720
Triglycerides, mg/dl	−0.031	0.654
Total cholesterol, mg/dl	0.073	0.286
HDL cholesterol, mg/dl	−0.081	0.230
LDL cholesterol, mg/dl	0.155	**0.022**
Estradiol, pg/ml	−0.162	**0.019**
Progesterone, ng/ml	−0.039	0.566
AUCg, g/dl x 120 min	−0.024	0.726
AUCi, U/l x 120 min	−0.018	0.794
AUCcp, ng/ml x 120 min	−0.013	0.844
HOMA-S%	−0.033	0.631
HOMA-B%	−0.014	0.843
OGIS, ml x min ^-1^ x m ^-2^	−0.043	0.556

*r*_*s*_, Spearman correlation coefficient.

Statistically significant *P* values are labeled with bold text.

## Discussion

Previous studies reported conflicting findings regarding Lp(a) concentrations in patients with T2D and its relationship with insulin resistance [7-14]. In addition, there are limited data currently available on Lp(a) in GDM, which is considered a pre-diabetic state. In the present study we demonstrated that at the time of GDM screening in late pregnancy women with NGT and GDM patients exhibit no difference in Lp(a) concentration. Furthermore, Lp(a) did not show any significant correlation with indices of insulin resistance and beta-cell function.

It has been demonstrated previously that insulin could lower Lp(a) levels and an inverse relationship between the incidence of diabetes and Lp(a) has been reported [8,13]. Interestingly, recent data suggested that only extremely high concentrations of Lp(a), above the threshold level of 46 mg/dl, are associated with less resistance to insulin [26]. Lp(a) levels are raised in pregnancy and increase during the course of pregnancy [27,28]. In most studies Lp(a) levels reached the maximum in the third trimester and after delivery Lp(a) concentrations returned to the baseline values [29]. Previous reports have shown different average Lp(a) levels at their peak in the last trimester of pregnancy. For example, one longitudinal study reported median peak Lp(a) values of 47,1 mg/dl [30]. Sattar et al. noted progressive rise in Lp(a) concentration during normal pregnancy with a maximal level of 27.0 mg/dl at 35 weeks gestation [31]. In our study subjects Lp(a) levels were just above the physiological concentrations, but did not reach the threshold of 46 mg/dl, which may explain the low correlation with the markers of insulin resistance.

It would be logical to assume that Lp(a), as a well established risk factor for CVD may be influenced by or may influence other known risk factors such as dyslipidemia or glucose intolerance. For example, blood glucose was correlated with the risk of coronary heart disease in the Framingham Offspring Study and was found to be an independent predictor for cardiovascular mortality [32,33]. However, there was no significant correlation between Lp(a) and glycemic biomarkers, fasting glucose, A1C and AUCg in our study group, which is in agreement with the findings from at least two other studies [34,35]. Small case-studies showed positive correlation between Lp(a) and HDL-C and a negative correlation between Lp(a) and TG [36,37]. In contrast, a low correlation between Lp(a) and lipids has been shown previously in a large prospective study of Lp(a) concentration and risk of T2D in initially healthy US women [11]. Similarly, Lp(a) did not show a significant correlation with other serum lipids in our study group, although we observed a significant positive relationship with LDL-C. This might be explained because LDL-C is included in the cholesterol contained in Lp(a) particles. The lack of correlation between Lp(a) and parameters of glycemia and lipid metabolism is not surprising given that systemic Lp(a) concentrations are highly genetically determined and only a few environmental or physiological factors have been shown to influence serum Lp(a) levels [38].

Consistent with data from others our women with GDM showed slightly lower levels of LDL-C than women with NGT [39]. However, in this study LDL-C postpartum levels were significantly higher in women who had GDM than in women who had NGT during pregnancy. Circulating LDL-C levels may influence the atherothrombotic impact of Lp(a) and this risk appears to be substantially attenuated among subjects in whom aggressive LDL-C lowering has occurred [40-44]. Similarly, as LDL-C and Lp(a) are correlated it could be possible that GDM blunted levels of Lp(a) during pregnancy. Therefore, future studies are needed to clarify the relationship between Lp(a) and insulin resistance in women with GDM and NGT postpartum.

## Conclusions

In summary, Lp(a) levels in pregnancy seem to be independent of the presence or absence of GDM. Additionally, our data suggest that the pathophysiological mechanisms of insulin resistance in GDM are not related to the Lp(a) concentration during pregnancy. Further studies are needed to explore the relationship between Lp(a) and insulin sensitivity in women with NGT and GDM postpartum.

## Abbreviations

AUCcp: Area under the curve of C-peptide; AUCg: Area under the curve of glucose; AUCi: Area under the curve of insulin; BMI: Body mass index; CRP: C-reactive protein; CVD: Cardiovascular disease; GDM: Gestational diabetes mellitus; HDL-C: High-density lipoprotein cholesterol; HOMA-B%: Homeostatis model assessments of beta–cell function; HOMA-IR: Homeostasis model assessment of insulin resistance; HOMA-S%: Homeostasis model assessment of insulin sensitivity; LDL-C: Low-density lipoprotein cholesterol; Lp(a): Lipoprotein(a); T2D: Type 2 diabetes; NGT: Normal glucose tolerance; OGIS: Oral glucose insulin sensitivity index; OGTT: Oral glucose tolerance test; TG: Triglycerides.

## Competing interests

The authors declare that they have no competing interests.

## Authors’ contributions

All authors participated in the design and coordination of the study. JT performed the statistical analysis and wrote the manuscript. AH, KL, JH, GH and AKW were involved in the interpretation of the analysis and critically revised the manuscript. All authors read and approved the final version of the manuscript.
